# Soil and forest floor respiration already acclimated to increasing temperatures in a mixed deciduous forest

**DOI:** 10.1186/s13717-025-00639-4

**Published:** 2025-09-01

**Authors:** Liliana Scapucci, Luana Krebs, Susanne Burri, Lukas Hörtnagl, Nina Buchmann

**Affiliations:** Department of Environmental Systems Sciences, ETH Zürich, Zurich, Switzerland

**Keywords:** Soil CO_2_ flux, Wild garlic, Respiration, Eddy-covariance, Acclimation, Climate change, Temperature

## Abstract

**Background:**

Forest ecosystems are in the spotlight for their potential to mitigate anthropogenic carbon dioxide (CO_2_) emissions through net photosynthesis. However, this mitigation potential can be counteracted by respiratory losses, e.g., from soils and the forest floor. With global warming, soil respiration (SR) rates are expected to increase, unless acclimation occurs. Using manual and automated chambers as well as a below-canopy eddy-covariance system, we quantified SR and forest floor net CO_2_ exchange (NEE_ff_) for 13 years throughout an 18-year study period (2006–2010, 2015–2016, 2018–2023) in a mixed deciduous forest ecosystem in Switzerland. We identified the contribution of environmental drivers for SR and NEE_ff_ using Extreme Gradient Boosting models and Shapley additive explanations (SHAP) analyses and assessed the long-term temperature sensitivity of SR and NEE_ff_.

**Results:**

Over the 18-year study period, soil temperature increased significantly and was the main driver of both SR and NEE_ff_, explaining over 50% of their temporal variability. Differences in drivers and magnitudes of SR vs. NEE_ff_ were only found in early spring, when the forest floor vegetation showed net CO_2_ uptake. Finally, we found no evidence that SR or NEE_ff_ (at mean annual temperatures) had increased between 2006 and 2023. Similarly, no significant change in the temperature sensitivity of SR and NEE_ff_ was observed.

**Conclusions:**

Combining multiple techniques to assess long-term responses of CO_2_ fluxes to environmental conditions with machine learning approaches enhanced our understanding of forest responses to climate change. Moreover, our findings suggest that soil and forest floor respiration already acclimated to warmer conditions, highly relevant for predicting future mitigation potentials of forest ecosystems.

## Background

Anthropogenic emissions of carbon dioxide (CO_2_) are still increasing, with large uncertainties of the land and ocean carbon sinks (Friedlingstein et al. 2023). Thus, political decisions concerning the mitigation of anthropogenic CO_2_ emissions rely on precise estimates of CO_2_ fluxes of the biosphere (IPCC 2022). Nature-based solutions, in particular, the potential of forest ecosystems to compensate anthropogenic CO_2_ emissions, are in the spotlight of current climate change mitigation debates (Anderegg et al. 2020; Keesstra et al. 2018). How forest soils respond to global warming is still not clear (Bossio et al. 2020). Although numerous studies have investigated the impact of rising temperatures on soil CO_2_ dynamics, they often show contradictory results (Carey et al. 2016; Hursh et al. 2017; Melillo et al. 2002). Since soils store more than three times the amount of carbon present in the atmosphere (Köchy et al. 2015), and global CO_2_ emissions from soils have been increasing between 1980 and 2010 (Hashimoto et al. 2023), this lack of understanding generates substantial uncertainties for climate change projections (Bond‐Lamberty et al. 2024).

The largest CO_2_ flux from terrestrial ecosystems to the atmosphere such as from forests is soil respiration (SR; Bond‐Lamberty et al. 2024; Davidson and Janssens 2006). SR is the result of all metabolic processes that release CO_2_ from the soil, i.e., heterotrophic respiration by microorganisms and soil fauna as well as autotrophic respiration of roots (Hogan et al. 2023; Ruehr and Buchmann 2010; Ryan and Law 2005; Zhang et al. 2020). It is well established that SR is positively related to temperature, but if and how this relationship, i.e., the temperature sensitivity of SR, has been changing over time with climate warming is less clear (Bond‐Lamberty et al. 2024; Davidson and Janssens 2006; Melillo et al. 2017). On the one hand, some studies reported a decrease in temperature sensitivity with increasing temperatures, either driven by water scarcity (Curiel Yuste et al. 2007; Jassal et al. 2008) or by decreasing C substrate availability (Davidson and Janssens 2006; Eberwein et al. 2015; Eliasson et al. 2005; Tang et al. 2019b). The increase in frequency and intensity of soil droughts, as projected for future climate conditions, can indeed limit metabolic reactions in the soil and thus SR (Manzoni et al. 2012; Ruehr et al. 2010). In addition, despite the temperature sensitivity of SR, a disequilibrium between organic matter decomposition and C supply via root exudates and litterfall can affect SR (Melillo et al. 2017). Some other studies however did not show a change in temperature sensitivity of SR over time (Contosta et al. 2013; Yan et al. 2019). Hence, long-term data sets are urgently needed to explore responses of SR to increasing temperatures due to climate change (Bond‐Lamberty et al. 2024; Chi et al. 2021).

Chambers are one of the most common methods to measure SR (Bond‐Lamberty et al. 2024). Automated chambers provide high temporal resolution measurements, but typically with limited spatial coverage (Janssens et al. 2001). In contrast, manual chambers capture the wider spatial representativeness (Cai et al. 2023; Jiang et al. 2020; Pennington et al. 2020), but with a lower temporal resolution (Bond‐Lamberty et al. 2024). Comparing fluxes from automated and manual measurements often showed similar responses to temperature (Ruehr et al. 2010), supporting the employment of either chamber type. In recent years, efforts to quantify net CO_2_ exchange of the forest floor or understory vegetation (NEE_ff_) with a below-canopy eddy covariance (EC) station (Aubinet et al. 2012) have become more frequent (Janssens et al. 2001; Law et al. 1999; Misson et al. 2007; Paul-Limoges et al. 2017). However, studies that compare flux measurements based on both techniques are scarce (Bastviken et al. 2022; Campioli et al. 2016).

In this study, long-term chamber measurements of soil respiration (SR) as well as eddy covariance (EC) measurements of the forest floor (NEE_ff_) in a mixed deciduous forest in Switzerland were investigated. Based on 13 years of data, collected during an 18-year study period between 2006 and 2023, this study aimed to (1) quantify CO_2_ fluxes from the soil and the forest floor, (2) identify the contribution of environmental drivers for SR and NEE_ff_, and (3) assess the long-term trend of SR and NEE_ff_ responses to increasing temperatures. We hypothesized that SR and NEE_ff_ have similar flux magnitudes and drivers that follow a seasonal trend. In addition, we expected to observe a decrease in temperature sensitivity driven by the increase in temperature over the past decades.

## Materials and methods

### Forest site

The Lägeren forest site (CH-Lae) is a mixed deciduous forest situated 20 km north-west of Zurich in Switzerland. The study area is located on the south facing ridge of the Jura mountain at 682 m asl, where two eddy covariance stations are placed (above-canopy at 47 m, below-canopy at 1.5 m). The forest is mainly composed of beech trees (*Fagus sylvatica* L.), other common species are Norway spruce (*Picea abies* L. Karst), ash (*Fraxinus excelsior* L.), Sycamore maple (*Acer pseudoplatanus* L.), European silver fir (*Abies alba* Mill.), and large-lived linden (*Tilia platyphyllos* Scop.)*.* The tree age ranges between approximately 52 and 155 years (Etzold et al. 2011). The soils of the study area are rendzic leptosols and haplic cambisols, with bedrock of limestone marl, sandstone, and transition zones (Ruehr et al. 2010). The annual mean temperature is 8.8 ± 1.3 °C (mean ± SD) and the annual mean precipitation is 831 ± 121 mm (mean 2005–2022; Scapucci et al. 2024). Over the years, there were no significant changes to the forest structure and the leaf area index (LAI) at the site (Scapucci et al. 2024). During spring, the main understory species, wild garlic (*Allium ursinum* L., height ~ 30 cm, LAI < 0.5; Paul-Limoges et al. 2017) blooms for approximately two months (March–April); some other herbaceous species and juvenile beech trees can be found at the site (<1% cover). The forest floor vegetation remains active until the tree canopy closes. Then only little radiation reaches the forest floor (Etzold et al. 2011; Ruehr and Buchmann 2010), thus impeding the growth of understory plants.

### Chamber measurements

#### Automated measurements 2006–2010

From January 2006 to December 2010, an automatic chamber (LI-8100-101; LI-COR Inc., Lincoln, NE, USA) was installed over a PVC collar (20.3 cm inside diameter, 11 cm high) about 50 m north-east of the Lägeren EC tower (for details, see Ruehr et al. 2010). Measurements of SR were taken automatically every 30 min during these five years, together with soil temperature (TS) at 5 cm depth and soil water content (SWC) at 10 cm depth (EC-20 Decagon Devices Inc., Pullman, WA, USA).

#### Manual survey measurements 2006–2007

Between January 2006 and December 2007, manual survey measurements of SR were performed at a regular basis, every 2–3 weeks between 10:00 and 16:00 (LI-8100 with closed LI-8100-103 chamber, LI-COR Inc, Lincoln, NE, USA). 16 PVC collars (19.6 cm inside diameter, 10 cm high, insertion depth 1.5 cm) were installed at the forest site, covering both soil types (as described in Ruehr et al. 2010). Soil temperature and soil moisture were measured next to each collar during each measurement campaign. Seasonality and magnitude of the SR rates measured manually during these two years matched those measured automatically very well (Ruehr et al. 2010).

#### Manual survey measurements 2022

In March 2022, 15 PVC collars (20 cm inside diameter, 13 cm high, insertion depth 2 cm) were installed following the earlier spatial design of Ruehr et al. (2010). SR was measured manually about every second week between 10:00 and 16:00 until June, using the same instrumentation as during 2006 and 2007. Similarly, the wild garlic leaves were cut at the beginning of the growing season (March), and eventual regrowth was cut prior the measurements. Between mid-May and end of June, the frequency of measurements was reduced to once a month since the tree canopy started to develop and completely shaded the forest floor. On each measurement day, each collar was measured once. In July 2022, however, the measurement frequency was increased due to a forecasted heatwave (see Scapucci et al. 2024). During this time, 10 out of 15 collars were measured twice a day (at 9:00 and 15:00) until the 11th of August 2022 when weather conditions reached the seasonal mean again (five of the collars could not be measured due to lateral air leaking caused by drought-induced soil cracks). Afterwards, measurements were performed again once a month for the same ten collars. Ancillary measurements of TS and SWC at 5 cm depth were performed with a temperature sensor (GTH 175 PT, GHM Messtechnik GmbH, Regenstauf, Germany) and a soil moisture sensor (HH2 Moisture Meter, Delta-T Devices, Cambridge, United Kingdom) close to each collar during each SR measurement.

In an additional experiment, during the spring months (from March to May 2022), 15 additional PVC collars were installed next to the other ones. The aim was to quantify respiration and net ecosystem CO_2_ exchange of the wild garlic on top of the soil (R_WG_ and NEE_WG_). For those measurements, the wild garlic leaves were not cut inside the collars. NEE_WG_ was measured with a 20 cm long home-built transparent cylinder attached to the LI-8100-103 chamber. Here, wild garlic leaves were sampled, dried, weighed and prepared for carbon concentration analysis with an elemental analyser (NA 2500; CE Instruments, Milan, Italy). From the number of leaves present in each collar and the average carbon concentrations of the leaves, we calculated the biomass and carbon pools (g m^−2^) of wild garlic.

### Eddy covariance and forest floor measurements

In 2014, a below-canopy EC (Aubinet et al. 2012) system was installed at the former location of the automated SR system (47°28′42.9"N and 8°21′27.6"E). Turbulent fluxes of CO_2_ were measured with a frequency of 20 Hz at 1.5 m height with an open path IRGA (LI-7500, LI-COR Inc., Lincoln Nebraska, USA) and a 3D sonic anemometer (R-350, Gill Instruments Ltd., Lymington, UK). The system measured the net forest floor CO_2_ exchange (NEE_ff_), i.e., respiration from plants and soil, and photosynthesis of the understory vegetation. Here, eight of the years with below-canopy flux data (2015, 2016, from 2018 to 2023) were used. The half-hourly CO_2_ fluxes (FC; μmol m^−2^ s^−1^) were calculated following community standards using the software *EddyPro* (v7.0.9, LI-COR Inc., Lincoln, NE, USA). CO_2_ molar density measurements obtained with the open-path IRGA were corrected for air density fluctuations (Webb et al. 1980). FC were corrected for high-pass and low-pass filtering effects to account for spectral losses (Horst 1997; Moncrieff et al. 2005). Then, the net CO_2_ exchange of the forest floor (NEE_ff_) was calculated as the sum of FC and the storage term (Greco and Baldocchi 1996), accounting for less than 1% change of FC. A common quality assessment method was applied to assure that only best quality data were considered to calculate NEE_ff_ (based on quality flags 0-1-2 system; Mauder and Foken 2006; Sabbatini et al. 2018; Scapucci et al. 2024), thus lowest quality data (flag = 2) were discarded. In addition, only best quality data (flag = 0) were used for nighttime measurements. Moreover, to assure that only measurements with adequate turbulence were considered, a friction velocity (u*) filter was applied. Seasonal u* thresholds were calculated for each year and season separately, based on REddyProc (Papale et al. 2006; Wutzler et al. 2018), to account for seasonal changes in canopy cover (Fig. 6 in appendix). Values below the u* thresholds were discarded (3.8% of the data). Around 32% of the measured data were finally used (21% daytime, 11% nighttime). Thus, missing data were gap-filled using the Marginal Distribution Sampling (MDS) method (Reichstein et al. 2005), of which more than 90% fulfilled the highest quality requirements. Following community conventions, negative NEE_ff_ values depict periods when photosynthetic CO_2_ uptake of the understory vegetation dominates the CO_2_ flux, while positive NEE_ff_ values represent situations when respiratory losses dominate the forest floor CO_2_ exchange. Next to the below-canopy EC station, photosynthetic photon flux density (PPFD; SQ-521, Apogee Instrument, Inc.) was measured at 2 m height, soil temperature (TS) and soil water content (SWC) were measured at multiple depths (5, 10, 20, and 50 cm) with the same sensor, first with a Decagon ECH2O EC-20 probes (Pullman, WA, USA; 2015–2020) and then with a TEROS 12_00007171 (METER group AG, NE, USA; 2020–2023).

**Fig. 1:**
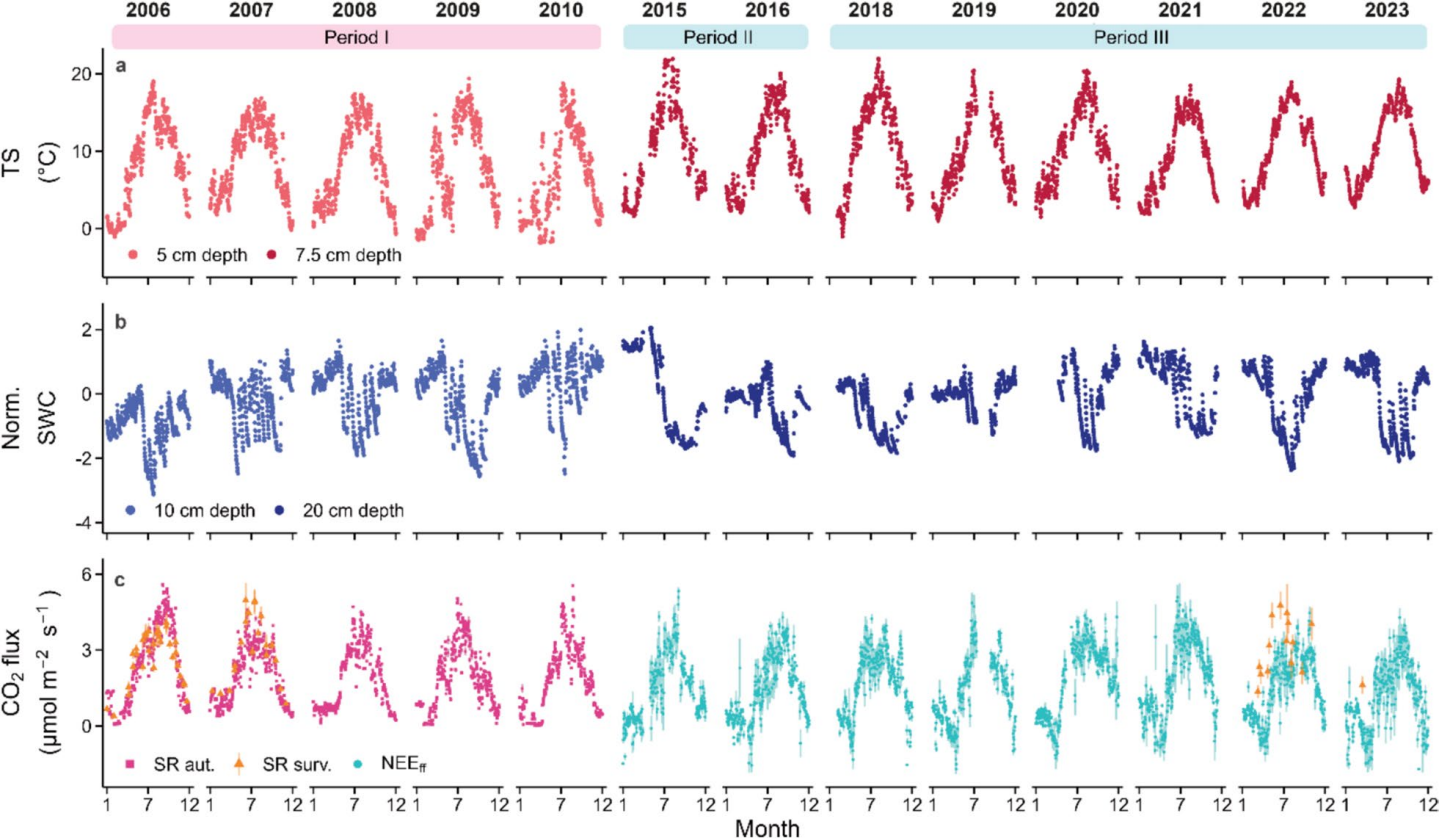
Soil temperature, soil water content and CO_2_ fluxes measured over 13 years. Daily mean soil temperature (TS; **a**), normalized soil water content (SWC; **b**) as well as soil respiration (SR; ± SE) and net CO_2_ exchange of the forest floor (NEE_ff_; ± SE) (**c**) measured at the Lägeren site for 13 years (2006–2010, 2015–2016, and 2018–2023) are given. Different colours identify different instruments (SR aut. and SR surv. represent automated and manual survey SR measurements, respectively) or soil depths (5, 7.5, 10, 20 cm depths). Three coloured bands mark the three different periods (time intervals) during which continuous measurements were collected

### Data analyses

SWC data, measured at 10 and 20 cm soil depths, were chosen since these depths had the longest and most complete time series and represented both, microbial and root activities. Data were normalized with a *z*-score transformation to account for different sensors employed over the 18 years. Moreover, soil temperature data, measured at 5 cm (2015–2023) and 10 cm (2015–2023) depths, were chosen and averaged over the whole period to reduce variability and to better represent topsoil conditions for microbial and root activities, hereafter referred to as at 7.5 cm soil depth. No difference was found between TS at both depths (*p* > 0.05; Fig. 7 in appendix). After daily mean calculations, annual mean, minimum and maximum values of SWC and TS were identified, and a Mann-Kandell trend analysis was applied to identify short-term (2006–2010; 2015–2023) and long-term (2006–2023) trends (Kendall 1948; Mann 1945). The slope of the trends was estimated with the Theil-Sen method (Sen 1968).

**Fig. 2:**
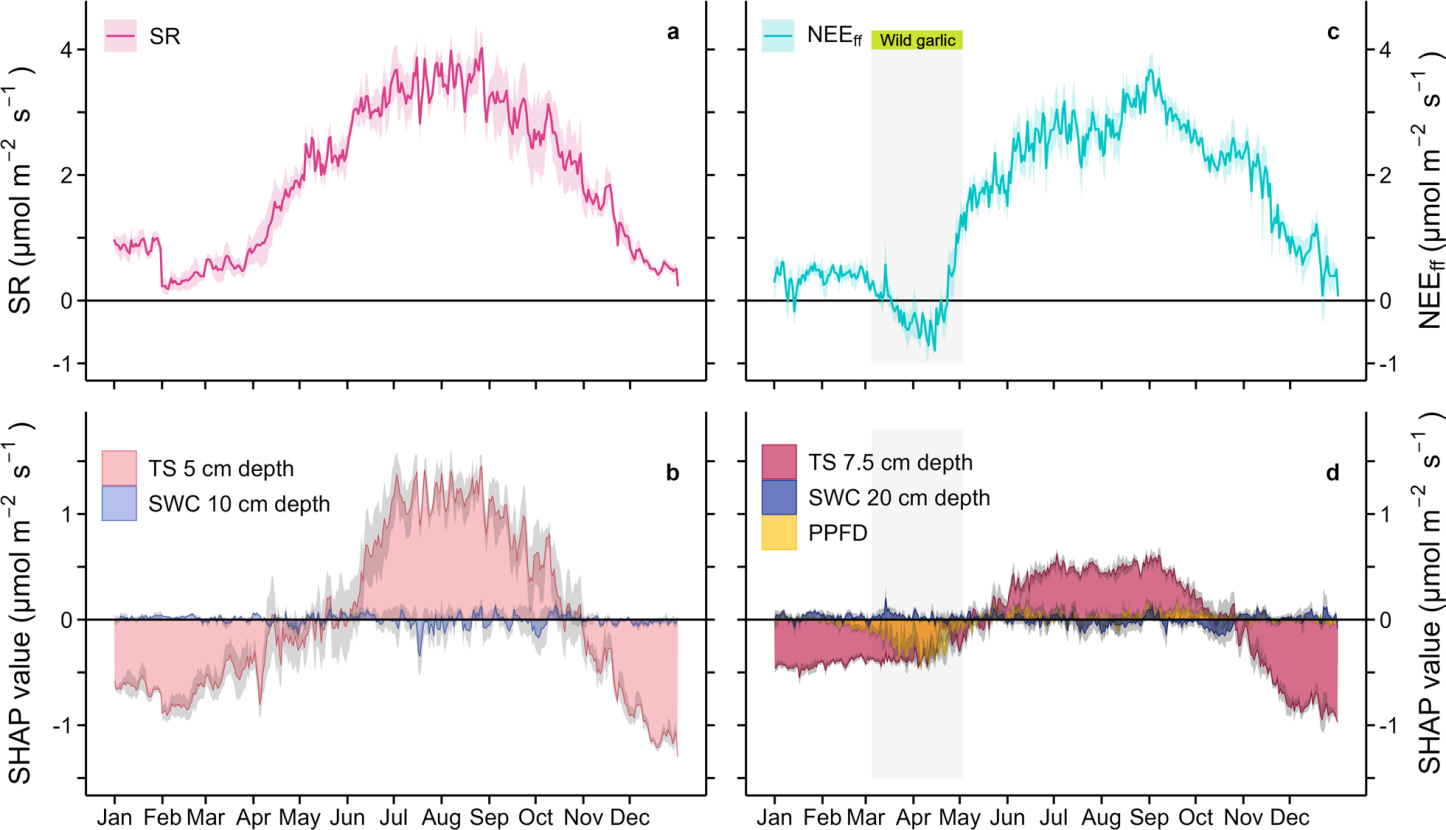
Drivers of soil respiration and net CO_2_ exchange of the forest floor. Mean daily soil respiration (SR; **a**) and net CO_2_ exchange of the forest floor (NEE_ff_; **c**) and contributions of environmental drivers to SR (**b**) and NEE_ff_ (**d**) are shown. Daily SR (±SE), measured with automated chambers, and daily NEE_ff_ (±SE), measured with the eddy covariance technique, were averaged per day for the years 2006–2010 and 2015–2023, respectively. Driver contributions, namely normalized soil temperature (TS) at 5 cm depth and normalized soil water content (SWC) at 10 cm depth for SR, normalized soil temperature (TS) at 7.5 cm depth as of 5 and 10 cm depth, normalized soil water content (SWC) at 20 cm depth, and photosynthetic photon flux density (PPFD) for NEE_ff_ are given by daily SHAP values (±SE indicated by the grey areas). A positive SHAP value indicates that the driver increased the respective flux compared to its mean, and vice versa

To estimate the marginal contributions of different drivers, TS and SWC for SR, and TS, SWC and PPFD for NEE_ff_, a TreeExplainer-based SHapley Additive exPlanations (SHAP) framework (Lundberg et al. 2020; Lundberg and Lee 2017) was used, combined with a tree-based ensembled learning eXtreme Gradient Boosting (XGB) model (Chen and Guestrin 2016; Scapucci et al. 2024). This method was chosen due to the high performance that the model can achieve, also considering collinearity among variables and allowing missing data, avoiding the need to gap-fill these missing data based on extra processing methods and functional assumptions (Aydin and Ozturk 2021; Chen and Guestrin 2016). For further details also refer to Scapucci et al. (2024). For SR, data measured between 2006 and 2009 were used as training data and 2010 data to validate the model (see Table 2 in appendix for model parameters and performance). NEE_ff_ data measured between 2015 and 2022 were used as training data and 2023 data to validate the model (see Table 2 in appendix for model parameters and performance). The features used to predict SR and NEE_ff_ were selected based on available data and commonly known drivers (i.e., TS and SWC). In detail, TS at 5 cm depth and SWC at 10 cm depth were normalized with a *z*-score transformation and daily means calculated, which were then used as drivers to predict SR. In accordance, the TS at 7.5 cm and SWC at 20 cm depths were normalized with *z*-score transformations (separately before and after 2020 due to a change in the sensor) and daily means were calculated. Normalized TS at 7.5 cm, normalized SWC at 20 cm, and PPFD (to address the effect of forest floor vegetation on NEE_ff_) were used as drivers to predict NEE_ff_. Two different XGB models were used to predict SR (XGB_SR_) and NEE_ff_ (XGB_NEEff_) flux measurements. The functions “xgboost” from the “xgboost” R package were used to train and validate the models, and “shap.values” and “shap.prep” from the R package “SHAPforxgboost” (Chen and Guestrin 2016) to obtain SHAP values for each of the drivers. Then, SHAP values (i.e., values corresponding to the importance of each feature in explaining variations in the response variable compared to the overall mean) were obtained for each day of each year and averaged per day across all years. The SHAP framework has become a widely used and powerful tool to investigate the temporal course of the feature (i.e., driver) importance. Thus, it allows to disentangle the temporal course of each feature (driver) contribution, highlighting the temporal variation of the effect of environmental variables, like soil temperature, on the response variable, like NEE_ff_ over a year.

**Table 1:** Soil temperature and soil water content values and trends over 13 years

	TS (°C)			SWC normalized		
	Min	Max	Mean	Min	Max	Mean
	5 cm depth			10 cm depth		
2006	−1.1	19.01	8.38	−3.12	0.24	−0.99
2007	−0.2	16.83	8.89	−2.48	1.99	−0.22
2008	−0.74	17.39	8.26	−1.91	1.65	0
2009	−1.52	19.37	7.86	−2.56	1.65	−0.36
2010	−1.9	18.75	6.51	−2.48	1.99	0.45
Trend	−	+	−	+	+	/
*p*-value	n.s.	n.s.	n.s.	n.s.	*	n.s.
	7.5 cm depth			20 cm depth		
2015	1.57	21.93	9.73	−1.7	2.06	0.13
2016	2.06	20.06	9.72	−1.91	0.99	−0.37
2018	−1.07	21.96	11.23	−1.84	0.58	−0.58
2019	0.96	20.4	8.69	−1.26	0.87	−0.01
2020	1.48	20.38	10.24	−1.76	1.49	0
2021	1.84	18.46	9.46	−1.33	1.63	0.167
2022	2.66	18.92	10.38	−2.37	1.17	−0.12
2023	2.71	19.26	10.44	−2.08	1.31	−0.12
Trend	+	−	+	−	+	/
*p*-value	n.s.	n.s.	n.s.	n.s.	n.s.	n.s.
2006–2023						
Trend	+	+	+	+	/	+
*p*-value	*	n.s.	*	n.s.	n.s.	n.s.

Annual minimum (Min), maximum (Max) and Mean values of TS at 5 cm (2006–2010) and at 7.5 cm (as average of TS at 5 and 10 cm depth; 2015–2023) were calculated for each year, based on daily mean values. Similarly, Min, Max and Mean values of normalized SWC at 10 cm (2006–2010) and 20 cm depth (2015–2023), expressed as *z*-scores, were calculated for each year, based on daily means. The results of the trend analyses for the respective periods are indicated as follows: positive and negative trends as well as no trends are shown with +, −, and/signs. The significance of the trends is indicated by the *p*-values (*p* > 0.05 n.s., *p* < 0.05*)

Partial dependence plots (i.e., SHAP values plotted against the corresponding feature values) were used to identify changes in the effects of TS and SWC on the response variables, fitted with a LOESS regression (span of 0.8; function “geom_smooth” of the R package “ggplot2”).

To assess the temperature sensitivity of SR and NEE_ff_, the equation Lloyd and Taylor (Eq. 1) was used (Lloyd and Taylor 1994).

1 $$SR (or {NEE}_{ff})= {R}_{ref}{e}^{{E}_{0}\left(\frac{1}{{T}_{ref}-{T}_{0}}-\frac{1}{TS- {T}_{0}}\right)}$$

where R_ref_ is the respiration at T_ref_, T_ref_ is the mean soil temperature of data used in the respective model fit, T_0_ = −46.02 °C as in the original Lloyd and Taylor model, E_0_ is the temperature sensitivity (K), and TS is the measured soil temperature at the site. The model was fitted on daily mean SR and NEE_ff_, and R_ref_, T_ref_, and E_0_ were determined for each year separately. In addition, the SR and NEE_ff_ at 10 °C TS (R_10_) was calculated from the Llyod and Taylor equation for each year. For the Lloyd and Taylor equation, both water limitations conditions and the wild garlic growing season period were excluded from the measurements. For the first one, SR and NEE_ff_ measurements when SWC was below the 10th lowest of the two periods (namely 2006–2010 and 2015–2023) quantile were removed (in accordance with knowledge from previous studies at the same site (Ruehr et al. 2010; Scapucci et al. 2024), and findings from the partial dependence plots about the effect of SWC on SR and NEE_ff_; Fig. 8 in appendix). For the second one, the wild garlic growing season was identified as the period during which the 8 years averaged daily mean NEE_ff_ was negative (namely between the 15th of March to 23 of April), with an addition of 10 days before the 15th of March and 10 days after the 23rd of April to account for seasonal shifts in the wild garlic growing season over the years. Thus, the wild garlic growing season was identified as the period between the 5th of March and the 3rd of May. During this period, photosynthetic processes dominate the CO_2_ exchange of the forest floor (thus NEE_ff_ decreases with increasing temperatures); while during the rest of the year, the CO_2_ exchange of the forest floor is dominated by soil respiration processes (thus NEE_ff_ increases with increasing temperatures). Since these two different conditions of the forest floor resulted in two opposite responses of NEE_ff_ to increasing temperatures, we focused the temperature sensitivity analysis on the period in which NEE_ff_ is dominated by SR, excluding the wild garlic growing season. Finally, the standard error of the parameters was calculated as the square roots of the diagonal elements of the covariance matrix obtained from the optimization process (inversion of the Hessian matrix). Similar magnitudes of SR and NEE_ff_ (Fig. 4) allowed for long-term analysis to identify trends in R_ref_, T_ref_, E_0_ and R_10_ over time (Mann–Kendall statistical test; Kendall 1948; Mann 1945; together with the Theil-Sen method; Sen 1968—to identify the slope).

**Fig. 3:**
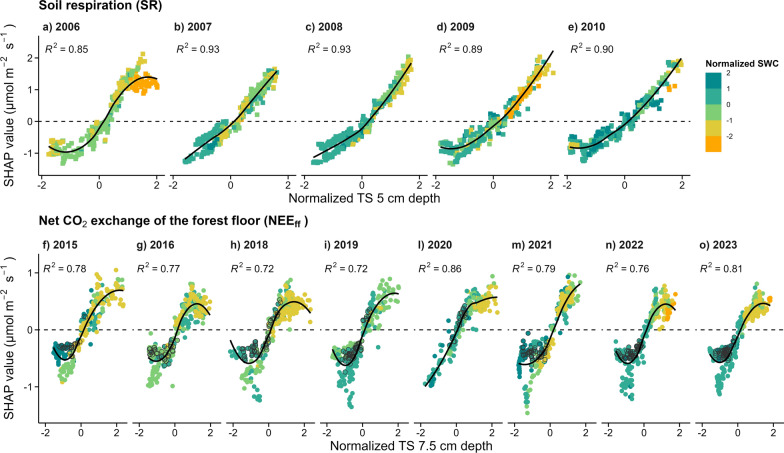
Effect of soil temperature on soil respiration and on net CO_2_ fluxes of the forest floor. Partial dependence plots of the marginal contribution of soil temperature (TS) to soil respiration (SR; measured with automated chambers between 2006 and 2010; **a**–**e**) and to net CO_2_ exchange of the forest floor (NEE_ff_; 2015–2023; **f**–**o**) during the study period. For each year, the corresponding SHAP values are plotted against normalized TS values (at 5 cm depth for SR, at 7.5 cm depth for NEE_ff_) enabling direct comparisons among years. A positive SHAP value indicates that TS increased the respective flux compared to its mean, and vice versa. The colour scale indicates normalized SWC values (at 10 cm depth for SR, at 20 cm depth for NEE_ff_), symbols with *black borders* represent the wild garlic season. Solid *black lines* describe the LOESS regression between normalized TS and the respective CO_2_ flux with a span of 0.8 (*R*^2^ is shown in the respective panels)

**Fig. 4:**
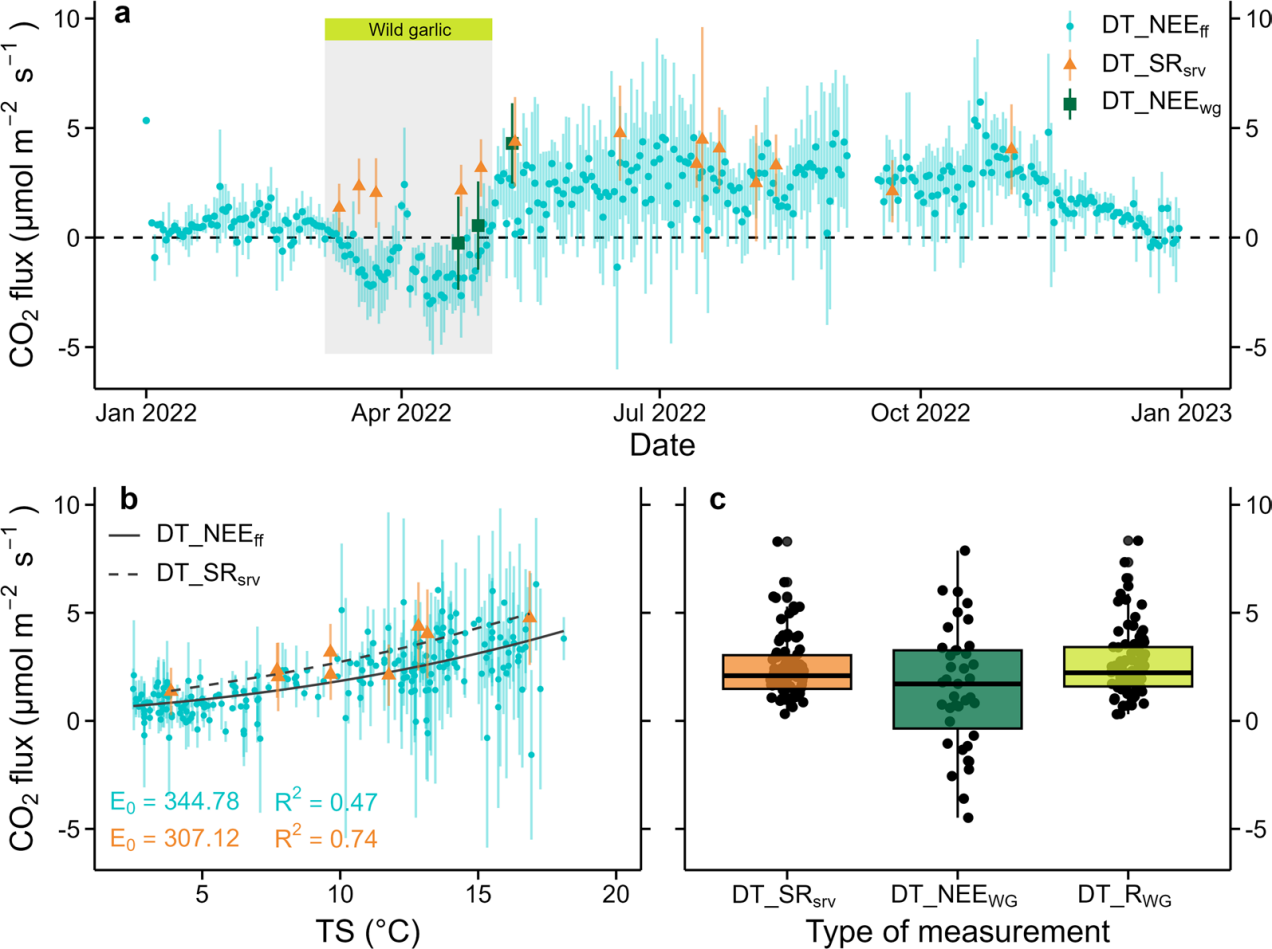
Comparison of CO_2_ fluxes measured with chambers and eddy covariance in 2022. Timeseries of mean daytime net CO_2_ exchange of the forest floor (DT_NEE_ff;_ between 10:00 and 16:00) and mean daytime SR measured with survey chambers (DT_SR_srv_; **a**) are given. Temperature relationships (based on the Lloyd and Taylor model) of DT_NEE_ff_ and DT_SR_ff_, temperature sensitivity (E_0_) and *R*^2^ values are shown as well (**b**). The wild garlic growing season was removed from DT_NEE_ff_ shown here. Three different measurement types, DT_SR_srv_, daytime NEE during the wild garlic season measured with transparent chambers (DT_NEE_WG_), and daytime dark respiration of the forest floor, i.e., soils and wild garlic understory, measured with opaque chambers (DT_R_WG_) are shown for springtime 2022 (from March to end of May 2022; **c**)

## Results

### Environmental conditions and CO_2_ fluxes

During all 13 years, TS at the Lägeren site showed clear seasonal courses at both soil depths (5 and 7.5 cm; Fig. 1a), with low TS during the winter months (January) and highest TS during summer (July). In contrast, normalized SWC showed a less pronounced seasonality, despite typically high SWC values during winter and spring (January–March) and low values during summer (July–August; Fig. 1b). SR rates, measured during 2006–2010 and 2022 with either automated and manual survey chambers, were lowest during winter (0–1.5 μmol CO_2_ m^−2^ s^−1^) and highest during summer (4–5 μmol CO_2_ m^−2^ s^−1^; Fig. 1c). This seasonality was also observed in the NEE_ff_ measurements (2015–2023). However, the wild garlic grwowing season was clearly visible, with NEE_ff_ fluxes between −1.5 and 0 μmol CO_2_ m^−2^ s^−1^ during March to May each year, indicating photosynthetic uptake by the understory vegetation overcompensating respiratory losses from the forest floor. Highest NEE_ff_ fluxes, representing respiration, were measured in July 2021 with 5 μmol CO_2_ m^−2^ s^−1^.

For the period 2006–2010, the lowest annual minimum TS at 5 cm soil depth was recorded in 2010 (−1.9 °C) and the highest maximum in 2009 (19.37 °C; Table 1). No significant trend (2006–2010) was observed in the minimum, maximum and mean annual TS at 5 cm depth. For the period 2015–2023, the average TS at 7.5 cm depth showed its annual minimum in 2018 (−1.07 °C) and the highest maximum in 2015 (21.93 °C; Table 1). In contrast to the earlier period, the warmest annual minima TS of 2.66 and 2.71 °C were higher than previously, and recorded in the most recent years, 2022 and 2023, respectively. This resulted in a significant (*p* < 0.01) overall increase in minimum TS and the mean annual TS (*p* < 0.05) for the entire period 2006–2023, while the maximum TS did not show a significant increase over time. SWC trends showed a significant increase in the maximum values at 10 cm depth (2006–2010), no significant changes were observed at 20 cm soil depth in the period 2015–2023 nor the entire period of 2006–2023 (Table 1).

### Driver analyses of SR and NEE_ff_

Mean daily SR rates (Fig. 2a) were very well predicted by the XGB_SR_ model (*R*^2^ = 0.80, RMSE = 0.09; Table 2 in appendix). TS at 5 cm depth was driving the seasonal dynamics of SR. Specifically, TS limited SR between November and April (SHAP values < 0; Fig. 2b) and increased SR between May and November (SHAP values > 0), which also coincided with the highest mean SR. In contrast, the effect of SWC was negligible overall (Fig. 2b). Also mean daily NEE_ff_ fluxes (Fig. 2c) were predicted well with the XGB_NEEff_ model (*R*^2^ = 0.65, RMSE = 0.09; Table 2 in appendix). Based on the SHAP analysis, TS was identified as the main feature mediating NEE_ff_ (Fig. 2d), followed by PPFD and SWC. Similarly to SR, TS constrained NEE_ff_ between November and May (SHAP values < 0; Fig. 2d) and enhanced NEE_ff_ between May and November (SHAP values > 0). PPFD showed pronounced effects on NEE_ff_ during the wild garlic season (March and April), increasing photosynthetic CO_2_ uptake of the understory vegetation (NEE_ff_ < 0 and SHAP values < 0; Fig. 2c, d) until the tree canopy started to close in May. Indeed, except for this short wild garlic season, PPFD had hardly any effect on NEE_ff_, which was mostly positive during that time, indicating that respiratory CO_2_ emissions of the forest floor to the atmosphere prevailed (Fig. 2c, d). In addition, the effect of SWC was much smaller compared to those of TS and PPFD, with noticeable impacts during early spring (May) when the wild garlic understory was active, but also later in the season (August, September, November).

This strong dependence of SR on TS was monotonic for most years (*R*^2^ > 0.85; Fig. 3a–e), except for 2006 (Fig. 3a) when low SWC levels at concurrently high TS values reduced SR. For NEE_ff_, such non-monotonic relationships between TS and respective SHAP values were observed for all eight years (*R*^2^ > 0.72; 2015–2023; Fig. 3f–o). When TS was still low and SWC high, increasing TS values were related to negative NEE_ff_ values, corresponding to the wild garlic growing in early spring. In contrast, when TS was high, a decrease in NEE_ff_ with increasing TS was observed, corresponding to lower-than-average SWC levels, as also observed for SR (Fig. 3a–e).

### Long-term trends in the temperature responses of SR and NEE_ff_

To assess potential changes in the temperature responses of SR and NEE_ff_ between 2006 and 2023, both measurement approaches (chambers as well as EC) and all years were used, but water-limited periods and wild garlic growing seasons were excluded from the analyses. The comparison was based on the good agreement found between daytime EC and survey SR measurements in 2022 (Fig. 4), both in terms of daytime averages (Fig. 4a) and responses to soil temperatures (Fig. 4b). In addition, we found no significant difference (*p* > 0.05) between daytime SR, daytime NEE_WG_ and daytime respiration of the wild garlic during the wild garlic growing season (R_WG_; Fig. 4c). At this time, the wild garlic understory vegetation had a leaf cover of 635 ± 288 leaves m^−2^, with 113 ± 65 g biomass m^−2^ and a carbon content of 47 g C m^−2^.

As expected, TS at 5 cm depth still explained most of the variation in SR (*R*^2^ of 0.62 to 0.94, *p* < 0.05; Fig. 9a–e in appendix), with more than 85% of the variance in SR during the earlier years (2006 to 2008; *R*^2^ > 0.85). In these three years, also higher temperature sensitivities (E_0_ > 380 K) were found than in the other years (2009–2010; E_0_ < 330 K; Table 3 in appendix), with the highest reference temperature found in 2007 (T_ref_ = 8.85 °C), together with the highest reference respiration (R_ref_ = 1.75 μmol m^−2^ s^−1^), while the lowest T_ref_ and R_ref_ were found in 2010 (T_ref_ = 6.36 °C) and 2008 (R_ref_ = 1.47 μmol m^−2^ s^−1^). During 2006 and 2010, the range in T_ref_ of SR was 2.49 °C, in R_ref_ 0.32 μmol m^−2^ s^−1^, and in E_0_ 154.2 K (Table 3 in appendix). TS also explained a large proportion of the variation in NEE_ff_ (*R*^2^ of 0.52 to 0.81, *p* < 0.05; Fig. 9 in appendix). The highest E_0_ was found in 2023 (378 K; Table 3 in appendix), which was also the year with the lowest R_ref_ (1.42 μmol m^−2^ s^−1^). In contrast, the highest R_ref_ was found in 2020 (R_ref_ = 2.71 μmol m^−2^ s^−1^), the year with the highest T_ref_ (T_ref_ = 11.94). During 2015 and 2023, the range was 2.68 °C in T_ref_, 1.29 μmol m^−2^ s^−1^ in R_ref_, and 228 K in E_0_, overall being higher than those for SR (Table 3 in appendix).

**Fig. 5:**
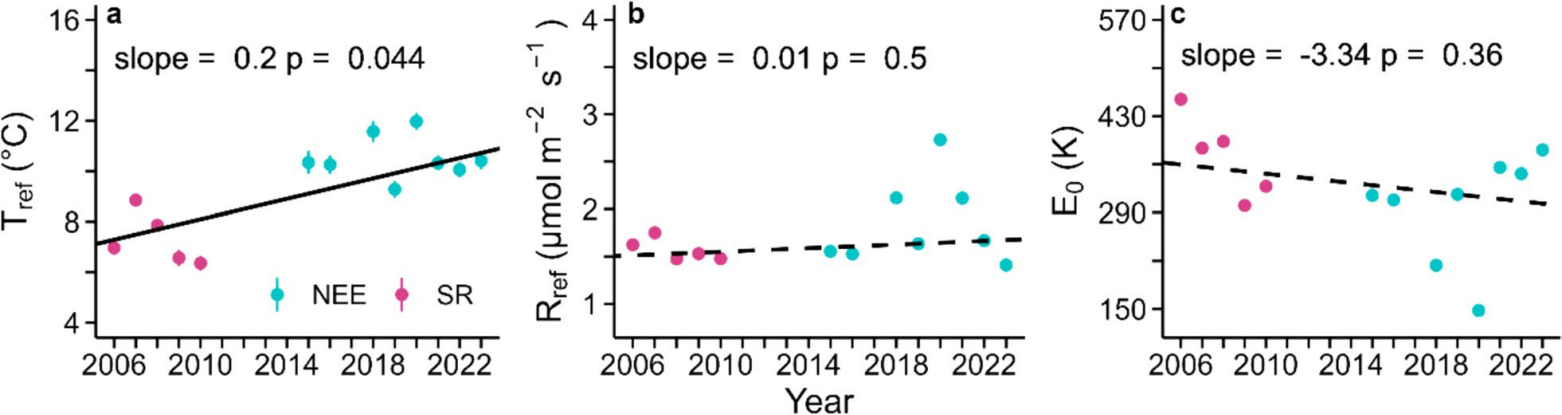
Long-term trends of T_ref_, R_ref_ and E_0_. Temperature responses of soil respiration (SR) and net CO_2_ exchange of the forest floor (NEE_ff_) are given as T_ref_ (**a**), R_ref_ (**b**), E_0_ (**c**) obtained from fitting the Lloyd and Taylor (1994) equation over time, i.e., from 2006 to 2023. The solid line indicates a significant trend, the dashed lines insignificant trends over time, calculated with Mann–Kendall trend analyses. Please note that SR and NEE_ff_ were measured with different approaches, yielding similar flux magnitudes (see Fig. 4)

Although a significant increase in Tref in the period 2006–2023 was found (slope of 0.2 °C year^−1^, *p* = 0.04; Fig. 5a), R_ref_ stayed constant (slope of 0.01 μmol m^−2^ s^−1^ year^−1^, *p* = 0.5; Fig. 5b), and E_0_ showed only a slightly negative trend (slope of −3.34 K year^−1^; *p* = 0.36; Fig. 5c). Overall, the overall variability among the years 2006 and 2023 was 5.6 °C for T_ref_, 1.32 μmol m^−2^ s^−1^ for R_ref_, and 306 K for E_0_ (Fig. 5).

## Discussion

Based on 13 years of SR and NEE_ff_ data, collected between 2006 and 2023, TS, mediated by SWC but also PPFD were identified as the main environmental drivers for SR and NEE_ff_, following a pronounced seasonal trend. Even though a long-term increase in TS was found, respiration at mean annual TS did not increase over time. Thus, no change in the temperature sensitivity of SR and NEE_ff_ was found, suggesting that soil and forest floor already acclimated to climate warming.

### Environmental conditions over time

Although no significant trend in TS was observed for the individual periods 2006–2010 and 2015–2023, the overall increase in annual minimum and annual mean TS observed for the entire period from 2006 to 2023 was significant (Table 1). Even if the comparison of TS measured at different depths could introduce a certain bias (TS at 5 cm depth for 2006–2010, average of 5 and 10 cm depth for 2015–2023), this would be rather small, since averaging a deeper (10 cm) and thus lower soil temperature in the last nine years compared to the first five years (5 cm depth) would tend to lower soil temperatures over time, the opposite of our finding. Moreover, we found no difference between TS measured at 5 vs. 10 cm soil depth in the last nine years (Fig. 7 in appendix), both depths and their average representing the variations of temperature in the biologically active topsoil well. Overall, the shift of TS in the topsoil over time was in fact remarkable.

Moreover, during the period 2006–2010, the annual minimum daily temperature was below 0 °C, while in recent years (2022 and 2023), it even exceeded 2 °C. Similarly, the annual mean temperature never reached 9 °C in the period 2006–2010, but it exceeded 10 °C three times during the period 2015–2023. These findings are in line with the projected increase in winter and mean annual temperatures for Europe (Copernicus Climate Change Service (C3S) 2023) and Switzerland (CH2018 2018) due to anthropogenic climate change. Such an increase in temperatures has considerable implications for the microbial communities and the forest floor vegetation (Dahl et al. 2023; Weigel et al. 2021), both in terms of physiological activities as well as for phenology, in particular since the increase in TS (estimated as 3.6 °C; Fig. 5) was much higher than that of measured air temperature at Swiss national level (2.8 °C increase since pre-industrial period 1871–1900; MeteoSvizzera 2024).

In addition to higher temperatures, low soil moisture conditions are expected to increase in Europe and Switzerland (CH2018 2018; Copernicus Climate Change Service (C3S) 2023; Grillakis 2019). However, no negative trend in SWC was found over time at our Lägeren mixed forest site (CH-Lae), maybe caused by different soil depths at which SWC was measured (10 cm depth in 2006–2010 vs. 20 cm depth in 2015–2023), by active understory vegetation modulating within canopy microclimate (Haesen et al. 2023; Lembrechts et al. 2020), as well as by high inter- and intra-annual variations, considerably masking any potential long-term trend (MeteoSvizzera 2024). For example, the lowest annual minimum and mean SWC values were found in those years in which heat waves and droughts occurred at the site, i.e., 2006 (Ruehr et al. 2010), 2018 and 2022 (Scapucci et al. 2024). Such extreme drought conditions have a strong impact on microbial and soil faunal communities, fine root activities in the soil (Bogati and Walczak 2022) as well as understory vegetation. Therefore, we recommend measuring SWC continuously at several depths, representing depths relevant for microbial activities (i.e., 2 to 10 cm depths) as well as for root activities (5 to 20 cm depths). Moreover, within-canopy, microclimate compilations as currently carried out (e.g., Lembrechts et al. 2022) need to be expanded and linked to fluxes to reliably model not only soil respiration but also forest floor CO_2_ fluxes.

### Drivers of SR and NEE_ff_ and their responses to temperature

The high importance of TS in driving seasonal changes of CO_2_ fluxes from the soil and the forest floor, already observed for SR at our Lägeren site (Ruehr et al. 2010), confirmed findings of other studies in a wide range of terrestrial ecosystems (i.e., Bahn et al. 2010; Carey et al. 2016; Curiel Yuste et al. 2007; Hogan et al. 2023). However, in a similar set up with chamber and below-canopy EC measurements, it was found that TS was the main driver of SR, but not of forest floor CO_2_ fluxes, due to the prevalent photosynthetic activity of mosses (Janssens et al. 2001). Those findings are in stark contrast with the NEE_ff_ observations at our Lägeren site, a mixed deciduous forest with an active understory vegetation composed of an early spring geophyte, wild garlic. Here, TS was the main driver of NEE_ff_, even during periods when the wild garlic covered the forest floor, and CO_2_ uptake of the forest floor was larger than CO_2_ emissions (Misson et al. 2007). During these wild garlic growing seasons, the effects of PPFD complemented the TS influence (Fig. 2d), nicely demonstrating the high importance of the photosynthetic activity of the forest floor vegetation (Chi et al. 2021; Law et al. 1999; Misson et al. 2007). These findings also underlined the different physiological responses of wild garlic to temperature compared to SR, suggesting that an increase in temperature would decrease NEE_ff_ during the wild garlic growing season due to CO_2_ uptake, while TS increased NEE_ff_ during the rest of the year. Hence, removing the wild garlic growing season period from the analysis of functional responses of CO_2_ fluxes to TS improved our understanding of long-term SR processes. Unfortunately, studies using concurrent measurements with chambers and below-canopy EC systems to account for the physiological responses of understory vegetation to temperature are still rare.

In contrast to TS, the overall effect of SWC on SR and NEE_ff_ was negligible. However, the responses of SR and NEE_ff_ to TS changed when SWC was low (Fig. 3). Under such dry conditions, the increase of SR and the NEE_ff_ with increasing TS was limited by soil water availability (Curiel Yuste et al. 2007; Rodríguez et al. 2023; Schindlbacher et al. 2012; Sun et al. 2019). In fact, low SWC can cause a reduction in the diffusion of soluble carbon substrates in the soil, resulting in lower microbial activities and thus in SR (Manzoni et al. 2012). Moreover, autotrophic respiration, a large component of both SR and NEE_ff_ (Ruehr and Buchmann 2010; Wang and Yang 2007), strongly depends on SWC as well (Tang et al. 2019a), and is further reduced by restrained carbon allocation from the canopy to the root system (Högberg et al. 2001; Ruehr et al. 2009). Hence, under low SWC conditions, a change in the sensitivity of respiratory fluxes to TS is to be expected (Jassal et al. 2008; Wang et al. 2014). Since we used SWC measured at 10 cm depth to determine drivers of SR and SWC at 20 cm depth for NEE_ff_, some effects at low SWC at shallower depths might have been masked. On the other hand, day-by-day variations in CO_2_ fluxes were well represented by the drivers used in our analyses, also under conditions of low SWC. Hence, intensity, frequency and length of water-limited periods have pronounced consequences for the responses of SR and NEE_ff_ to changes in temperature and thus on forest ecosystems (Scapucci et al. 2024), increasing the uncertainty for predicting responses of CO_2_ fluxes from forests in the future, particularly for those with a highly active understory vegetation (Bond-Lamberty et al. 2024; Hursh et al. 2017; Zhang et al. 2024).

### Long-term trends in the responses of CO_2_ fluxes to temperature

**Fig. 6:**
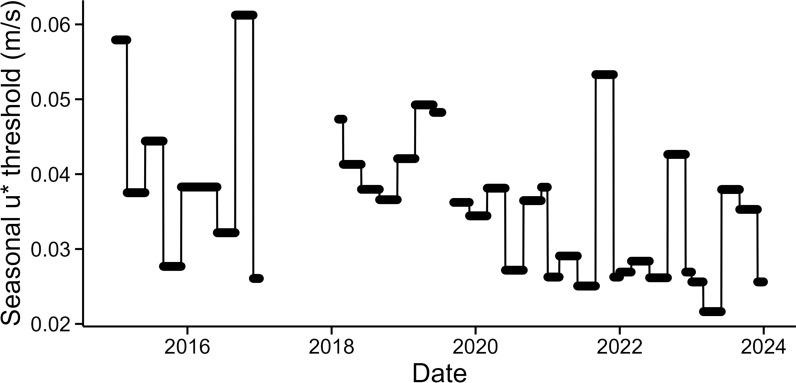
Friction velocity (u*) seasonal filter. The *black thick lines* represent the seasonal u* thresholds used to filter data when turbulence was too low. Thus, data below these u* thresholds were discarded from the below-canopy eddy covariance data set

**Fig. 7:**
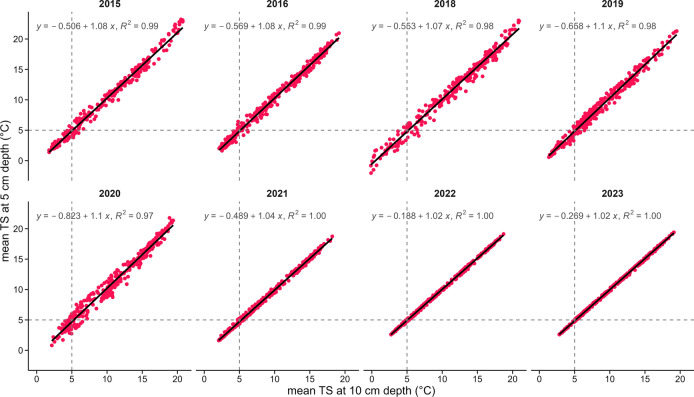
Soil temperature measured at different soil depths. Comparison of soil temperature (TS) at 5 vs. 10 cm depth over the years. A linear regression was done between daily mean TS at 5 and 10 cm depth for each year, the corresponding equation and *R*^2^ are given in each panel. The *dashed lines* indicate the 5 °C TS measured at 5 and 10 cm depth

**Fig. 8:**
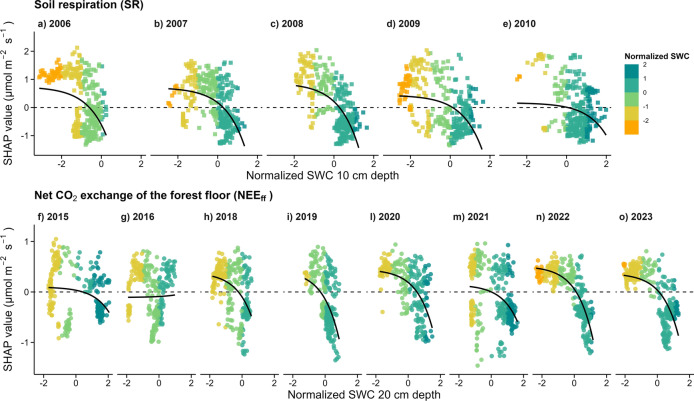
Effect of soil water content on the CO_2_ fluxes. Partial dependence plots of the marginal contributions of soil water content (SWC) to soil respiration (SR; measured with automated chambers, 2006–2010; **a**–**e**) and to net CO_2_ exchange of the forest floor (NEE_ff_; 2015–2023; **f**–**o**) during the study period. For each year, the corresponding SHAP values are plotted against normalized SWC values (at 10 cm depth for SR, at 20 cm depth for NEE_ff_) enabling direct comparisons among years. A positive SHAP value indicates that SWC increased the respective flux compared to its mean, and vice versa. The colour scale indicates normalized SWC values (at 10 cm depth for SR, at 20 cm depth for NEE_ff_). Solid black lines describe the LOESS regression between normalized SWC and the respective CO_2_ flux with a span of 0.8

**Fig. 9:**
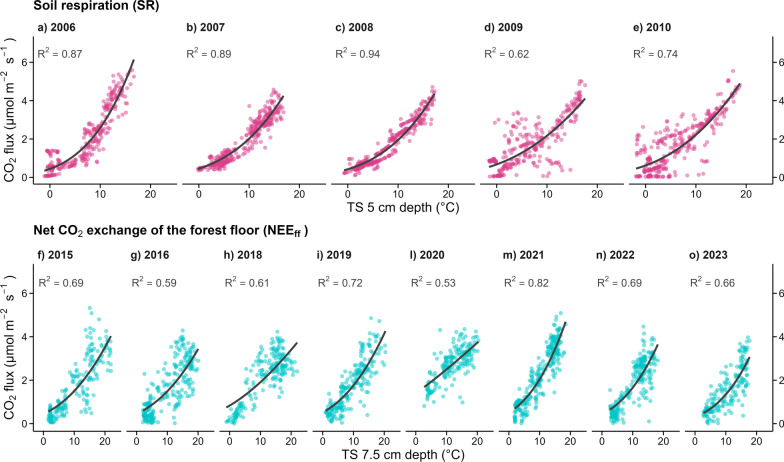
Lloyd and Taylor model applied on the CO_2_ fluxes over 13 years. Temperature response of soil respiration (SR; **a**) and net CO_2_ exchange of the forest floor (NEE_ff_; **b**) over the 13 years of the study. The Lloyd and Taylor equation was fitted against soil temperature (TS) for each year separately, excluding water-limited periods and the wild garlic season (*solid black lines*; for details, see "Data analyses" section). The goodness of the fits is given by *R*^2^, all relationships were significant (*p* < 0.05). TS at 5 cm soil depth was used for SR, average TS at 7.5 cm depth for NEE_ff_

Focusing on conditions without water-limitations and periods outside the wild garlic growing seasons, we combined two measurement approaches (chambers and EC system) to assess long-term trends in the responses of SR and NEE_ff_ to increasing soil temperatures between 2006 and 2023, i.e., during an 18-years study period (Table 1, Fig. 5). In agreement with earlier studies (e.g., Janssens et al. 2001; Lucas-Moffat et al. 2018; Myklebust et al. 2008; Shi et al. 2022), below-canopy EC and chamber measurements matched well (Fig. 4a,b), particularly since SR was the major component of NEE_ff_ outside the wild garlic seasons (Figs. 1, 4a, c). Small differences between EC and chamber measurement approaches could be related to intrinsic uncertainties of either technique (Janssens et al. 2001). In our study, the effect of such errors was avoided by using measured, best quality fluxes only. Another potential bias when comparing different measurement approaches could be the spatial heterogeneity of the forest floor. However, already Ruehr et al. (2010) found strong agreement between many surveys and few automated chamber SR measurements at our site, also in response to TS. Similarly, we found that between 50 and 90% of the variance in SR and NEE_ff_ were explained by TS. Moreover, even during the wild garlic growing seasons, we found that dark respiration of wild garlic measured on forest soil did not differ significantly from SR without vegetation, clearly indicating that the respiratory activity of wild garlic did not significantly increase SR. The low understory LAI of <0.5 (Paul-Limoges et al. 2017) likely explained this observation. Moreover, we found that the wild garlic C pool represented only 0.3% of the aboveground C pool at the site (Etzold et al. 2011), with no significant change in forest structure over time (Scapucci et al. 2024). Another challenge of measuring below-canopy EC fluxes can be low turbulence during nighttime, represented by low friction velocity (u*; Janssens et al. 2001; Wutzler et al. 2018). However, at our forest site, turbulence was adequate during both daytime and nighttime (Figure 6 in appendix), supporting our approach of combining high quality, reliable data from different measurement approaches to assess TS in regulating CO_2_ fluxes from the soil and the forest floor over the long-term.

**Fig. 10:**
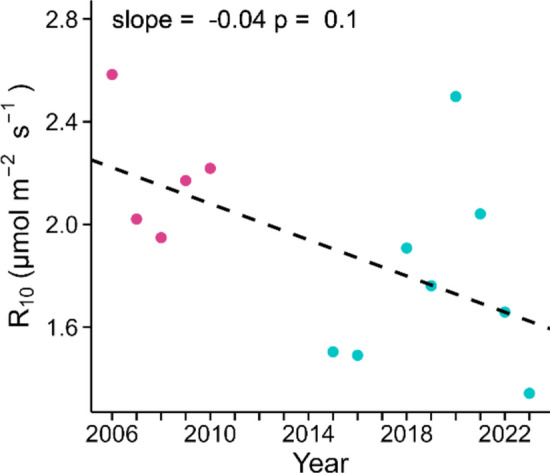
Long-term trend of R_10_ over time. Trend of soil and forest floor respiration at 10 °C soil temperature (R_10_) for each of the years (R_10_ obtained by the Llyod and Taylor equation). The dashed line indicates a non-significant trend over time, calculated with Mann–Kendall trend analyses. Slope and *p*-value are shown

The large inter-annual variations in the parameters T_ref_, R_ref_ and E_0_, found during the period 2006–2023, suggested that temperature sensitivities and CO_2_ flux responses to temperature were highly complex and thus difficult to predict (Bond‐Lamberty et al. 2024; Carey et al. 2016; Davidson and Janssens 2006; Hursh et al. 2017). With warmer temperatures, an increase in microbial activity is expected (Melillo et al. 2017; Tang et al. 2019b), which can lead to decreasing soil carbon stocks over time (Davidson and Janssens 2006). Indeed, in mid-latitude temperate forests such negative feedback effects were observed: SR first increased with warmer temperatures but then decreased because soil carbon stocks and thus carbon substrate availability for microorganisms decreased (Melillo et al. 2017). In accordance, R_ref_, i.e., the respiration at T_ref_, did not increase at the Lägeren site, although T_ref_ increased (Fig. 5): instead, R_ref_ stayed constant between 2006 and 2023. Similarly, R_10_ showed a non-significant decreasing trend (Fig. 10 in appendix). These findings clearly suggested that soil and forest floor respiration at our mixed forest site already acclimated to climate warming. This interpretation was also supported by the non-significant change of E_0_ over time, the temperature sensitivity, at the Lägeren site, supporting earlier experimental studies (i.e., Carey et al. 2016; Melillo et al. 2017). Such an acclimation response can be driven by carbon substrate depletion (Janssens et al. 2010; Melillo et al. 2011), but also by adjustments in the metabolic rates of microbial communities and roots to higher temperatures (Davidson and Janssens 2006). We hypothesize that at our site, the observed acclimation in SR and NEE_ff_ was most likely driven by the microbial community, since the contribution of understory dark respiration to forest floor respiration was very small (Fig. 4c). Overall, these findings from our long-term field study at the Lägeren forest suggested that annual soil and forest floor CO_2_ emissions did not increase in the past 18 years and that future CO_2_ emissions from both soil and forest floor will stay in a similar order of magnitude, unless extreme events, like forest disturbances (Rodríguez et al. 2023), extreme rain (Lee et al. 2002) or extreme drought (Scapucci et al. 2024) events, change the microbial community and related respiration rates.

## Conclusions

The interplay of increasing TS, seasonally changing PPFD, and different SWC levels generate complex responses in CO_2_ fluxes from soils and the forest floor, mostly dominated by SR. These responses can best be assessed in the field with a below-canopy eddy covariance system which measures at higher temporal resolution and integrates over larger areas than chambers, although such below-canopy systems are still rather scarce. Long-time series of data, even collected with different instruments at times, combined with machine learning methods, are a robust resource to detect intra- and inter-annual variations of fluxes and to improve our understanding of forest ecosystem responses to climate change. Their relevance would even increase if measurements were collected consistently, e.g., at identical soil depths over time, especially in the biologically active zone of the topsoil. Our study highlighted that—despite increased soil temperatures—acclimation responses during the last 18 years limited CO_2_ emissions from soils and the forest floor at our mixed deciduous forest, most likely dominated by microbial acclimation. If and to what extent such acclimation also occurred at other sites and how these acclimation responses will react to extreme events, e.g., to drought recurrences, remains to be seen.

## Data Availability

The datasets generated and/or analysed during the current study are openly available in the online ETH research collection (10.3929/ethz-c-000783003).

## Appendix A

See Figs. 6, 7, 8, 9, 10 and Tables 2, 3

**Table 2:** Parameters used in the eXtreme Gradient Boosting Training (XGB) models

Parameters	SR	NEE_ff_
*eta*	0.4	0.2
*max_depth*	8	8
*gamma*	0.07	0.04
*subsample*	0.9	0.98
*colsample_bytree*	0.86	0.86
*nrounds*	200	200
*early_stopping_rounds*	10	10
Performance		
*R*^2^	0.8	0.65
RMSE (μmol m^−2^ s^−1^)	0.09	0.084
Features	Position	
TS normalized	5 cm depth	7.5 cm depth
SWC normalized	10 cm depth	20 cm depth
PPFD	/	2 m

XGB models were used to predict soil respiration (SR) and net CO_2_ exchange of the forest floor (NEE_ff_) and respective performance indicators (*R*^2^ and RMSE) on unseen test data reported. Features used in the XGB for SR and NEE_ff_ models are also shown (*eta* = controls the learning rate; *max_depth* = maximum depth of the tree; *gamma* = minimum loss reduction for the next partition on the tree’s leaf nod; *subsample* = fraction of columns to be subsampled when constructing each tree; *colsampe_bytree* = subsample ratio for the tree’s column; *nrounds* = maximum number of the boosting iteration; *early_stopping_rounds* = number of rounds after which to stop if the performance of the model does not improve)

**Table 3:** Parameters obtained from the Lloyd and Taylor model over 13 years

Year	T_ref_ (°C)	R_ref_ (μmol m^−2^ s^−1^)	E_0_ (K)
SR			
2006	6.97 ± 0.29	1.63 ± 0.05	454 ± 17
2007	8.85 ± 0.26	1.75 ± 0.06	383 ± 18
2008	7.86 ± 0.27	1.47 ± 0.05	393 ± 16
2009	6.57 ± 0.33	1.53 ± 0.05	300 ± 15
2010	6.36 ± 0.30	1.48 ± 0.05	328 ± 13
NEE_ff_			
2015	10.47 ± 0.44	1.57 ± 0.06	315 ± 18
2016	10.30 ± 0.36	1.54 ± 0.05	308 ± 19
2018	11.69 ± 0.41	2.12 ± 0.05	214 ± 15
2019	9.25 ± 0.33	1.63 ± 0.05	315 ± 18
2020	11.95 ± 0.32	2.71 ± 0.05	150 ± 15
2021	10.31 ± 0.29	2.10 ± 0.05	359 ± 17
2022	10.11 ± 0.30	1.69 ± 0.05	343 ± 22
2023	10.45 ± 0.33	1.42 ± 0.06	378 ± 31

Changes in temperature response of soil respiration (SR) and net CO_2_ exchange of the forest floor (NEE_ff_) during the 13 years of the study. Parameters obtained from the Lloyd and Taylor equation (Eq. 1) are given, applied for each year separately (excluding water-limited periods and the wild garlic season): T_ref_ is the mean soil temperature, R_ref_ is the reference respiration at T_ref_, and E_0_ is the temperature sensitivity

## Abbreviations

CO_2_: Carbon dioxide
SR: Soil respiration
NEE_ff_: Net CO_2_ exchange of the forest floor
DT_NEE_WG_: Daytime NEE of wild garlic
DT_R_WG_: Wild garlic dark respiration
DT_SR_srv_: Daytime SR measured with a survey chamber
TS: Soil temperature
SWC: Soil water content
PPFD: Photosynthetic photon flux density
T_ref_: Reference temperature
R_ref_: Reference respiration
E_0_: Temperature sensitivity
R_10_: Respiration at a soil temperature of 10 °C
